# Opposite roles of Kindlin orthologs in cell survival and proliferation

**DOI:** 10.1111/cpr.13280

**Published:** 2022-07-20

**Authors:** Irina Zhevlakova, Luyang Xiong, Huan Liu, Tejasvi Dudiki, Alieta Ciocea, Eugene Podrez, Tatiana V. Byzova

**Affiliations:** ^1^ Department of Neurosciences Lerner Research Institute, Cleveland Clinic Cleveland Ohio USA; ^2^ Department of Inflammation and Immunity Lerner Research Institute, Cleveland Clinic Cleveland Ohio USA; ^3^ Present address: CVRC, Simiches Research Center Massachusetts General Hospital, Harvard Medical School Boston Massachusetts USA; ^4^ Present address: Hondros College of Nursing Westerville Ohio USA

## Abstract

**Objective:**

It is unclear why adhesion‐dependent cells such as epithelium undergo anoikis without anchorage, while adhesion‐independent blood cells thrive in suspension. The adhesive machinery of these cells is similar, with the exception of Kindlin orthologs, Kindlin 2 (K2) and Kindlin 3 (K3). Here we address how Kindlins control cell survival and proliferation in anchorage‐dependent and independent cells.

**Material and Methods:**

To demonstrate the opposite roles of Kindlin's in cell survival we utilized in vivo and in vitro models and K3 and K2 knockdown and knockin cells. We used human lymphocytes from the K3 deficient patients in tumour model, K3 knockout and knockin macrophages and K2 knockout and knockin MEF cells for experiments in under conditions of adhesion and in suspension.

**Results:**

Depletion of K3 promotes cell proliferation and survival of anchorage‐independent cells regardless of cell attachment. In contrast, the absence of K2 in anchorage‐dependent cells accelerates apoptosis and limits proliferation. K3 deficiency promotes human lymphoma growth and survival in vivo. Kindlins' interaction with paxillin, is critical for their differential roles in cell anchorage. While disruption of K2‐paxillin binding leads to increased apoptosis, the lack of K3‐paxillin binding has an opposite effect in adhesion‐independent cells.

**Conclusion:**

Kindlin ortologs and their interaction to cytoskeletal protein paxillin define the mechanisms of anchorage dependence. Our study identifies the key elements of the cell adhesion machinery in cell survival and tumour metastasis, proposing possible targets for tumour treatment.

## Graphical abstract

Cell anchorage is critical for tissue morphogenesis and protection against dysplasia and cancer metastasis. Only transformed and circulating haematopoietic cells thrive without anchorage. The adhesive machinery of anchorage‐dependent and ‐independent cells is similar, with the exception of Kindlin orthologs, Kindlin 2 (K2) and Kindlin 3 (K3). Our study reveals paradoxically opposite roles of K2 and K3 in cell survival despite their identical functions in cell adhesion. K3 deficiency promotes human lymphoma growth and survival. K3 deficiency protects anchorage‐independent cells from apoptosis and promotes their proliferation, while K2 deficiency triggers apoptosis and diminishes proliferation in anchorage‐dependent cells. We further demonstrate that Kindlins' effects on proliferation rely upon the interaction between Kindlin and paxillin in both, anchorage‐dependent and ‐independent cells.

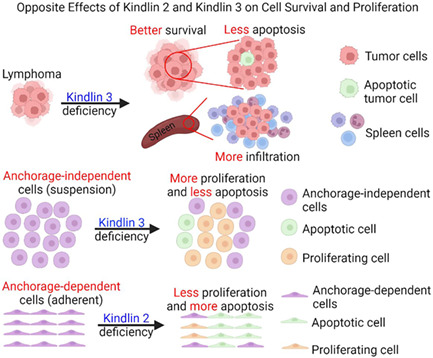

## 1 INTRODUCTION

Most cells in the body are programmed to survive and function only in an optimal and appropriate extracellular matrix (ECM) microenvironment; therefore, their well‐being is inseparable from their anchorage (adhesion) to a familiar ECM milieu.^1^ ‘Homeless’ cells that are detached from their original ECM undergo anoikis, programmed cell death due to the lack of adhesion.^2^, ^3^, ^4^ An exception to this is ECM‐independent leukocytes and other haematopoietic cells. Leukocytes are anchorage‐independent cells travelling in blood vessels and lymphatic tracts and are programmed not to interact with the ECM unless they are activated and immobilized as a result of ligand binding either within the blood clot or on the surface of the inflamed endothelium.^5^, ^6^, ^7^

The ECM recognition machinery, that is, the integrin repertoire and the content of integrin complexes known as the adhesome, is nearly identical in anchorage‐dependent and ‐independent cells. The transmembrane integrin heterodimers interconnect ECM and actin cytoskeleton via a dynamic set of adapters, including talins, Kindlins, vinculin, paxillin (PXN) and focal adhesion kinase (FAK)^8^ amongst many others.^9^, ^10^ One of the major features that distinguish adherent cells from suspension cells is the Kindlin family, which consists of adaptor proteins containing FERM and pleckstrin homology (PH) domains.^11^, ^12^, ^13^ Kindlin 1 (K1) and Kindlin 2 (K2) are expressed in many anchorage‐dependent cells, from fibroblasts to muscle cells, where they enable integrin‐dependent adhesion, migration and proliferation, thereby controlling the overall integrity of the tissue.^11^ K1 and K2 are embedded within adhesion complexes providing perpetual cell attachment. In contrast, Kindlin 3 (K3), which is exclusively expressed in anchorage‐independent haematopoietic cells, is not normally engaged in any interactions.^14^ K3 binding to integrins and other adaptors is inducible and rapid, leading to integrin activation and immediate binding to the ECM, which terminates their anchorage independence.^1^, ^12^ Thus, not merely the type of Kindlins, but also the functional consequences of their binding to other proteins might define the anchorage‐independent nature of haematopoietic cells.

Since K2 expression ensures adhesion and survival of anchorage‐dependent cells, abnormally high K2 expression might enable anoikis resistance. Anoikis resistance is detrimental to tumour cell survival and metastasis.^2^ Considering the critical role of Kindlins in adhesomes and transduction of ECM signals across the membrane, they might be able to regulate the anoikis of tumour cells and tumour survival. Indeed, in several malignancies such as kidney cancers, *Fermt2* (encoding K2) is either mutated or amplified compared to normal cells, which has been linked to poor patient survival (www.cbioportal.org). In contrast, analysis of *Fermt3* (encoding K3) in patients with haematological cancers revealed the presence of mutations in 2% of non‐Hodgkin lymphomas and deep deletions in B‐lymphomas and leukaemia as compared to nonmalignant cells (www.cbioportal.org). Overall, while K2 expression is beneficial for the survival of transformed cells, K3 might play a growth‐limiting role (www.cbioportal.org).

The molecular mechanisms responsible for these associations are unclear. The studies on the causative role of K2 and K3 in transformed cells are extremely controversial.^15^, ^16^, ^17^, ^18^, ^19^, ^20^, ^21^, ^22^, ^23^, ^24^, ^25^, ^26^, ^27^, ^28^, ^29^, ^30^, ^31^, ^32^ A number of counteractive functions have also been assigned to other members of the adhesomes, including but not limited to PXN family members, FAK and others.^8^, ^33^, ^34^, ^35^ This is likely due to the extensive use of knockouts and knockdown approaches, which abolish multiple protein–protein interactions with opposing consequences and can, therefore, render such studies mechanistically inconclusive. Since K2 and K3 isoforms represent the main distinction amongst integrin binding partners of adherent versus haematopoietic (suspension) cells, we aimed to define and compare the role of these two Kindlins in cell survival and proliferation.

In this study, we show that a lack of K3 supports human lymphoma cell growth and survival. While both K2 and K3 are required for cell adhesion, K2 and K3 have opposing functions in apoptosis and proliferation of anchorage‐dependent and ‐independent cells; while K3 deficiency restrains apoptosis and promotes proliferation of anchorage‐independent cells, K2 deficiency triggers anoikis and diminishes proliferation of anchorage‐dependent cells. However, these opposing effects of K3 and K2 on proliferation both involve an interaction between Kindlin and PXN.

## 2 RESULTS

### 2.1 K3 deficiency supports human lymphoma growth and survival

**FIGURE 1:**
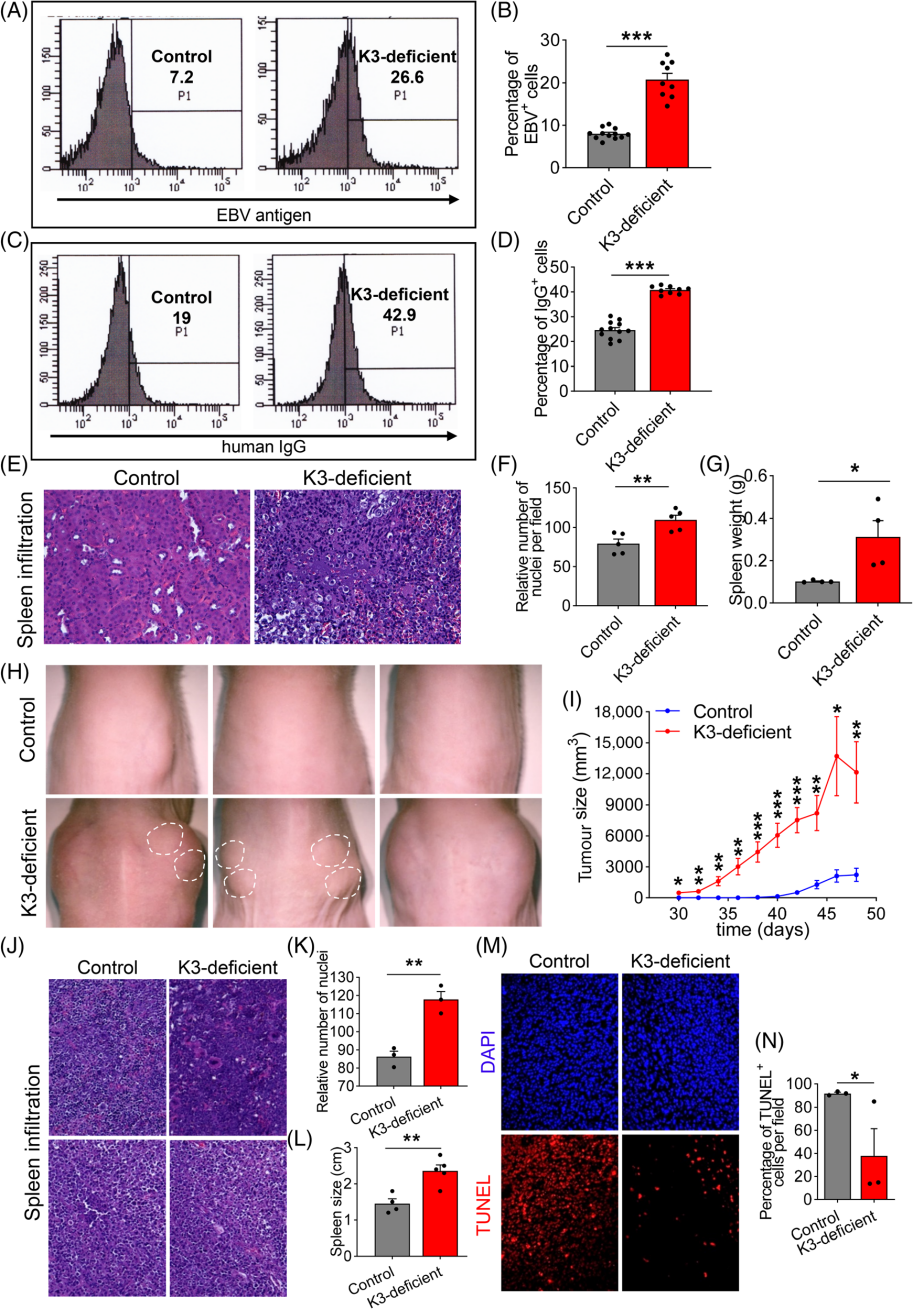
K3 deficiency supports the growth of human lymphoma cells. (A,C) Flow cytometry analyses of EBV antigen expression (A) and human IgG expression (C) in cells from the abdominal flush of severe combined immune deficiency (SCID) mice intraperitoneally injected with immortalized human peripheral lymphocytes with normal (Control) or deficient K3 (K3‐deficient) expression. Numbers represent percentages of cells in corresponding quadrants. (B,D) Bar graphs showing percentages of EBV^+^ (B) or human IgG^+^ (D) cells in flow cytometry analyses from A and C. *N* = 12 and 9 for control and K3‐deficient, respectively. (E) Representative H&E staining micrographs of spleen tissue sections from SCID mice intraperitoneally injected with immortalized human peripheral blood lymphocytes with normal or deficient K3 expression. (F) Quantification of the relative numbers of nuclei in images from E showing significantly more cells in the K3‐deficient group. *N* = 5. (G) Bar graph showing heavier spleens in the K3‐deficient group compared with the control group. *N* = 4. (H) Representative images of tumour growth in nude mice after subcutaneously injection with immortalized human peripheral blood lymphocytes expressing normal or deficient K3. Note that tumours in K3‐deficient group were of larger size and multinodular structure (dashed lines). (I) Line chart showing much bigger and faster subcutaneous tumours growth in the K3‐deficient group compared with the control group. *N* = 10 and 12 for control and K3‐deficient, respectively. (J) Representative H&E micrographs of spleen tissue sections from the control and the K3‐deficient groups (same mice as in H). (K) Bar graph showing an increased number of nuclei in the K3‐deficient groups compared with the control group in images from J. *N* = 3. (L) Spleen size was significantly larger in the K3‐deficient group compared with the control group (same mice as in H and J). *N* = 4 and 5 for control and K3‐deficient, respectively. (M) Representative TUNEL staining images of xenograft tumour tissue sections in the control and K3‐deficient groups. (N) Quantification of TUNEL^+^ cells in images from M shows fewer apoptotic cells in tumours from the K3‐deficient group compared with the control group (same mice as in H and J). *N* = 3. All values are mean ± SEM. Statistical significance was determined by Student's *t*‐test (N, D, F, G, K, L and N) or two‐way ANOVA (I).

Oncogenic signals promote anoikis resistance which favours tumour metastasis and survival^2^; therefore, we used Epstein–Barr virus (EBV)‐transfected K3‐deficient human peripheral blood lymphocytes (absence of Kindlin 3 protein expression was confirmed by Western blot^36^) as a tumour model to delineate the effects of K3 deficiency on tumour growth. The EBV provides an oncogenic signal and immortalizes the lymphocytes, making them a model for human lymphoma.^36^, ^37^ EBV‐immortalized control and K3‐deficient human peripheral blood lymphocytes were injected into the abdominal cavity of severe combined immune deficiency (SCID) mice. Cells obtained from the abdominal cavity were analysed by flow cytometry for EBV antigen and human IgG expression. The results show that the abdominal flush from K3‐deficient group contained three‐fold more human cells compared with that from control group (Figure 1A–D). These immortalized tumour cells also infiltrated and colonized the spleen. The spleens from K3‐deficient group were characterized by more severe tumour infiltration, as evidenced by a higher cell count and three‐fold larger spleen (Figure 1E–G). This result implies that K3 deficiency supports tumour cells survival not only in the abdominal cavity (presumably anchorage‐independently), but also favours their metastasis to other organs.

Next, we injected these tumour cells subcutaneously into nude mice. Tumours in the K3‐deficient group are significantly larger and grow faster than those in control group (Figure 1H,I). Again, more severe spleen infiltration was seen in the K3‐deficient group (Figure 1J,L), which might be attributable to better survival of K3‐deficient tumour cells in both the subcutaneous and blood compartments compared with control tumour cells (Figure 1M,N). Together, these results suggest that K3 deficiency promotes tumour growth in potentially anchorage‐independent and ‐dependent in vivo settings.

### 2.2 K3 deficiency suppresses apoptosis of anchorage‐independent cells

**FIGURE 2:**
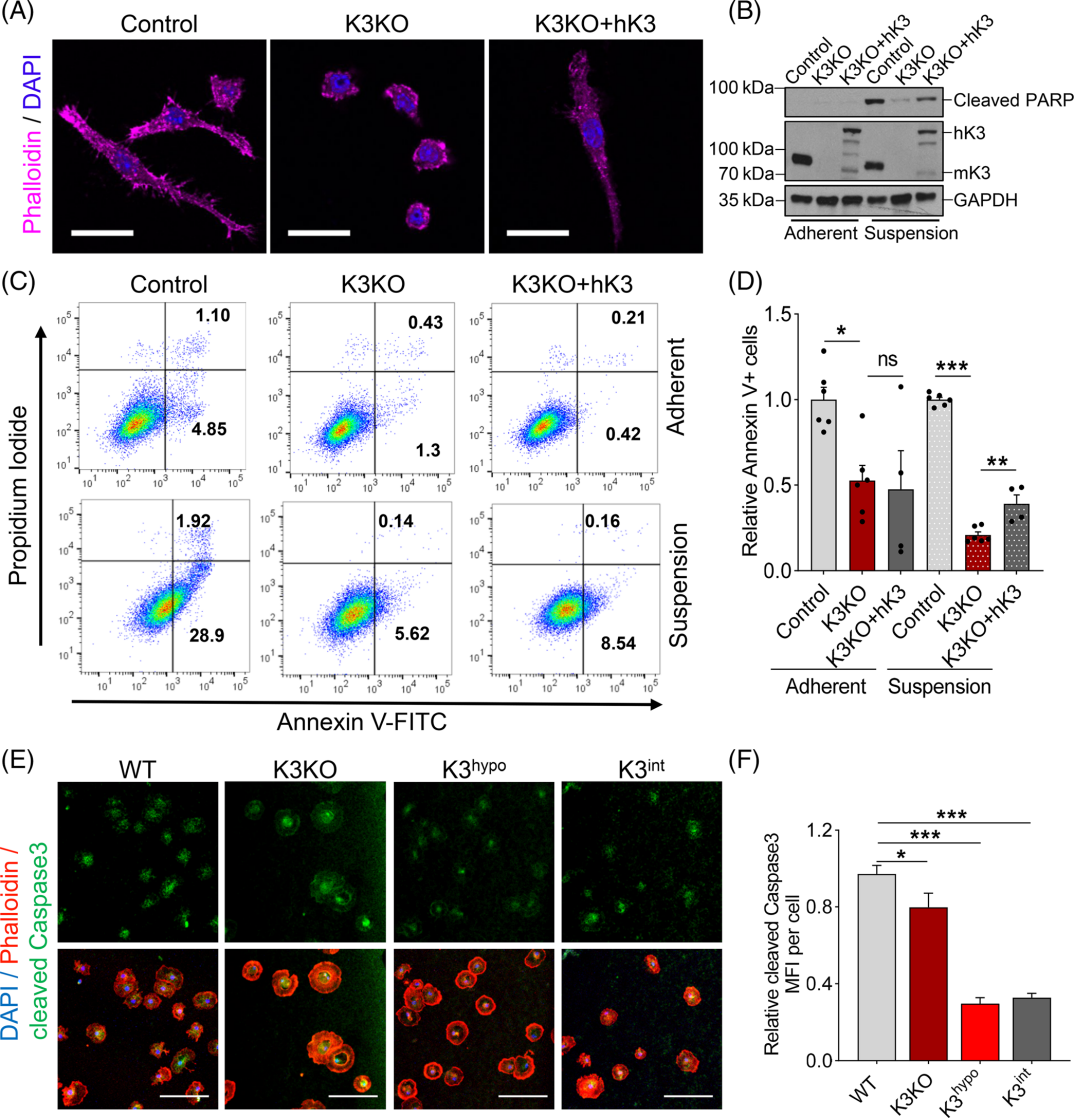
K3 deficiency protects anchorage‐independent cells from apoptosis. (A) Representative confocal images of control, K3KO (K3 knockout by CRISPR‐Cas9) and K3KO + hK3 (K3 knockout with re‐expression of human K3) Raw 264.7 macrophages stained with Phalloidin. Note the normal and stretched macrophages changed into an abnormal and round shape with decreased spreading after K3 deletion, and this change was reversed by re‐expression of human K3. Scale bars are 20 μm. (B) Western blot analysis showing the expression of cleaved PARP (an anoikis marker) in control, K3KO and K3KO + hK3 Raw 264.7 macrophages in adherent or suspension cultures. (C) Flow cytometry analyses of Annexin V expression by control, K3KO and K3KO + hK3 Raw 264.7 macrophages in adherent or suspension cultures. Numbers represent percentages of cells in corresponding quadrants. (D) Quantification of Annexin V^+^ apoptotic cells in C. Data were normalized to the control group. ‘ns’ means not significant. *N* = 4. (E) Representative confocal images of primary murine microglia stained with Phalloidin and cleaved Caspase3. K3KO microglia were generated by Cre‐loxP technology. K3^hypo^ and K3^int^ microglia expressed low level (~15%) or full level of mutant K3 with QW mutations at the integrin‐binding site, which resulted in diminished integrin binding. Scale bars are 100 μm. (F) Quantification of Caspase3 mean fluorescent intensity (MFI) per cell in images from e showing less cleaved Caspase3 expression in K3‐deficient (K3KO, K3^hypo^ and K3^int^) microglia compared with wild‐type microglia. *N* = 3. Data were normalized to the control group. All values are mean ± SEM. Statistical significance was determined by one‐way ANOVA.

K3 is nearly exclusively expressed in anchorage‐independent haematopoietic cells, however, its role in apoptosis is unclear. K3 was knocked out in Raw 264.7 macrophages by CRISPR‐Cas9 gene editing to produce a stable K3 knockout (K3KO) cell line. The lack of the Kindlin3 protein expression was confirmed by Western blot.^38^, ^39^ The absence of K3 completely abolishes cell adhesion, which is rescued by re‐expression of human K3 (hK3) (Figure 2A). The loss of K3 suppresses anoikis of K3KO macrophages in suspension culture as evidenced by the downregulation of cleaved PARP (an anoikis marker) and this effect is reversed by re‐expression of hK3 (Figure 2B). In contrast, K3 deficiency has no effect on macrophages in adherent culture (Figure 2B). Flow cytometry analyses of cell apoptosis by detecting Annexin V further strengthen our results and shows that deletion of K3 in macrophages reduces apoptosis with more than ~5‐fold decrease in suspension culture. Re‐expression of K3 partially reverses this effect in suspension culture rather than adherent culture (Figure 2C,D), suggesting that this effect is indeed anchorage‐independent.

We also confirmed the protective effect of K3 deficiency on apoptosis in microglia which is another typical anchorage‐independent cell. K3‐deficient (K3KO, K3^hypo^ and K3^int^) microglia were described previously.^39^ K3 expression is knocked out by Cre‐loxP gene editing in K3KO microglia. K3^hypo^ and K3^int^ microglia express low level (~15%) and full level of mutant K3 with QW mutations at the integrin‐binding site, respectively, as confirmed by q‐PCR and Western blot.^40^ Thus, the K3 in these cells is lacking either in amount or functional activity. Apoptosis was reduced by as much as 60% in K3‐deficient microglia compared with wild‐type microglia (Figure 2E,F). These results imply that while K3 is required for adhesion, K3 deficiency protects primary cells from apoptosis.

### 2.3 K3 deficiency promotes proliferation of anchorage‐independent cells in a K3‐PXN interaction‐dependent manner

**FIGURE 3:**
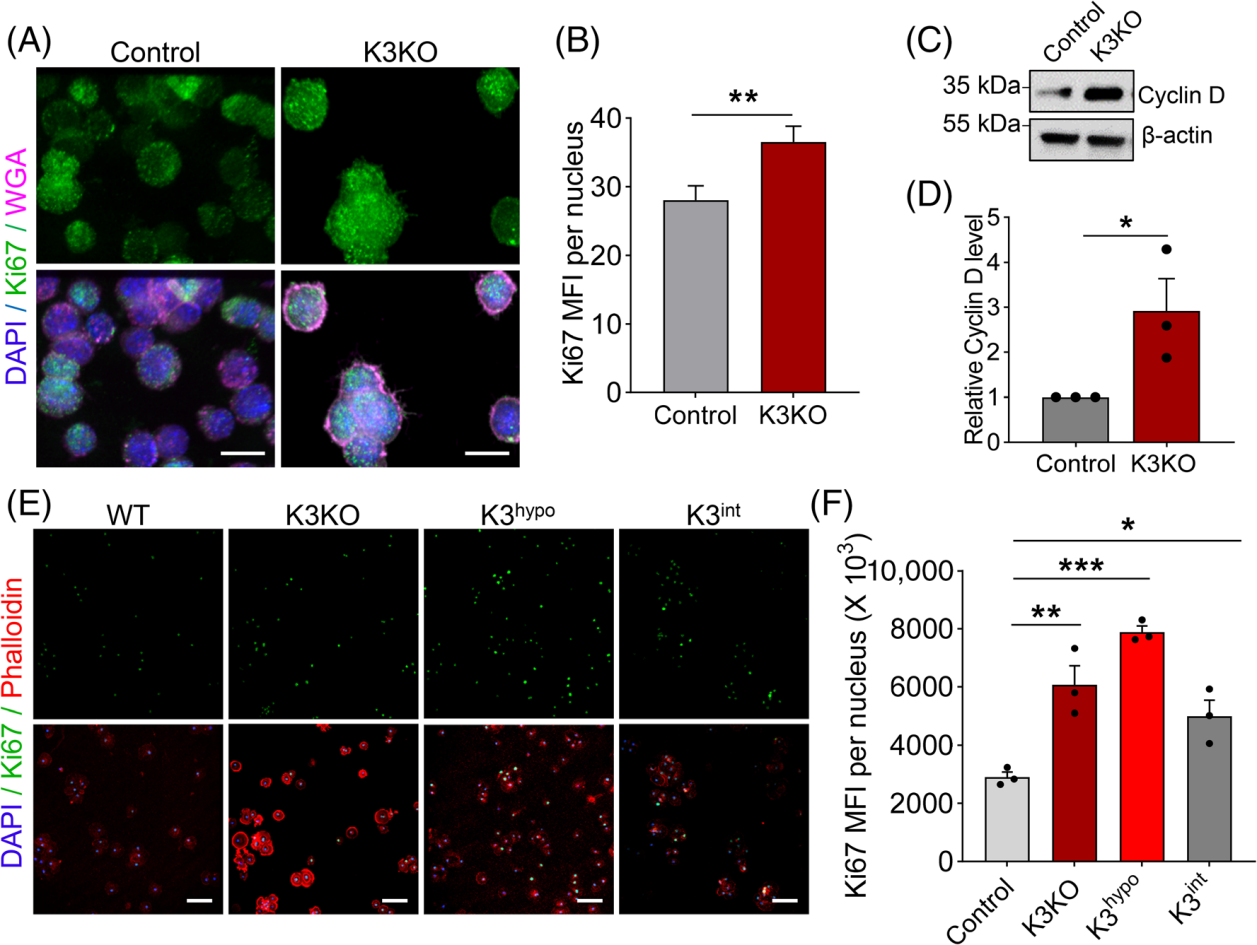
K3 deficiency promotes proliferation of anchorage‐independent cells. (A) Representative confocal images of control and K3KO Raw 264.7 macrophages stained with Ki67 and WGA. Scale bars are 10 μm. (B) Quantification of Ki67 mean fluorescent intensity (MFI) in images from A showing significantly more Ki67 expression in K3KO macrophages. *N* = 3. (C) Western blot analysis of Cyclin D expression in control and K3KO macrophages. (D) Quantification of Cyclin D protein expression in C revealing significantly higher Cyclin D expression in K3KO macrophages. *N* = 3. (E) Representative confocal images of wild‐type and K3‐deficient (K3KO, K3^hypo^ and K3^int^) primary murine microglia stained with Ki67 and Phalloidin. Scale bars are 100 μm. (F) Quantification of Ki67 mean fluorescent intensity (MFI) in images from e showing significantly higher Ki67 expression in K3‐deficient microglia compared with wild‐type microglia. *N* = 3. All values are mean ± SEM. Statistical significance was determined by Student's *t*‐test (B and D) or one‐way ANOVA (F).

We next examined K3's effect on cell proliferation. The lack of K3 resulted in increased proliferation of macrophages leading to a substantial growth advantage over control cells based upon expression of the proliferation marker Ki67 (Figure 3A,B). This was further confirmed by the increased protein expression of Cyclin D, a proliferative marker, in K3KO macrophages (Figure 3C,D). Different K3‐deficient primary microglia (K3KO, K3^hypo^ and K3^int^) also showed growth advantages over wild‐type microglia (Figure 3E,F), as was seen in macrophages. Collectively, these data indicate that a lack of K3 stimulates the growth of anchorage‐independent cells.

**FIGURE 4:**
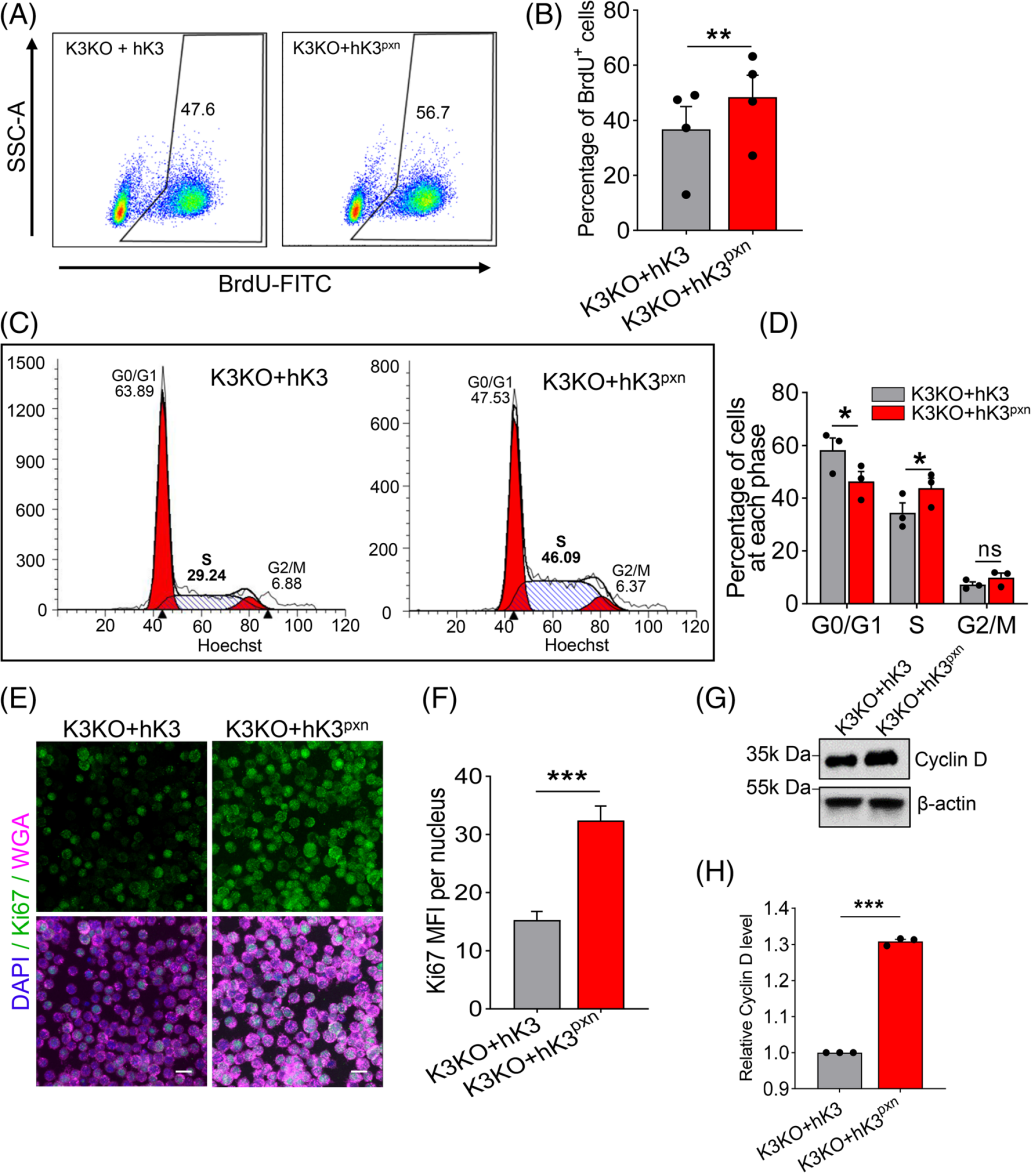
Promotion of cell proliferation by K3 knockout is dependent upon K3‐PXN interaction. (A) Flow cytometry analysis of BrdU incorporation by K3KO + hK3 and K3KO + hK3^pxn^ Raw 264.7 macrophages. K3KO + hK3^pxn^ represents K3KO cells with re‐expression of human K3 carrying mutations at the PXN‐binding site. (B) Quantification of flow cytometry analysis in A showing significantly more BrdU^+^ cells in K3KO + hK3^pxn^ group compared with the K3KO + hK3 group. (C) Flow cytometry cell cycle analysis of the K3KO + hK3 and K3KO + hK3^pxn^ Raw 264.7 macrophages. Numbers represent the percentages of cells in each phase. *N* = 4. (D) Quantification of cell cycle analysis showing a decreased number of cells in the G0/G1 phase and more cells in the S phase in the K3KO + hK3^pxn^ group compared with the K3KO + hK3 group. *N* = 3. (E) Representative confocal images of K3KO + hK3 and K3KO + hK3^pxn^ Raw 264.7 macrophages stained with Ki67 and WGA. Scale bars are 10 μm. (F) Bar graph showing significantly higher Ki67 mean fluorescent intensity (MFI) in K3KO + hK3^pxn^ cells. *N* = 3. (G) Western blot analysis of Cyclin D protein expression in K3KO + hK3 and K3KO + hK3^pxn^ Raw 264.7 macrophages. (H) Bar graph showing increased Cyclin D expression in K3KO + hK3pxn cells. *N* = 3. All values are mean ± SEM. Statistical significance was determined by Student's *t*‐test (B, F and H) or two‐way ANOVA (D).

To assess whether the interaction between K3 and PXN might account for the role of K3 in cell proliferation, we re‐expressed hK3 and hK3^pxn^ (human K3 carrying mutations at the PXN‐binding site) in K3KO macrophages at similar protein levels^40^Flow cytometry analyses of BrdU incorporation showed that the K3KO + hK3^pxn^ group had a higher proportion of cells entering and progressing through the S (DNA synthesis) phase of the cell cycle compared with the K3KO + hK3 group (Figure 4A,B). This result was then verified in flow cytometry cell cycle analysis revealing fewer cells in the G0/G1 phase and more cells in the S phase in the K3KO + hK3^pxn^ group compared with the K3KO + hK3 group (Figure 4C,D). Furthermore, fluorescent staining of the proliferative marker Ki67 and western blot analysis of the Cyclin D expression yielded similar results (Figure 4E–H), supporting the conclusion that the K3‐PXN interaction underlies the beneficial effect of K3 deficiency on proliferation of anchorage‐independent cells.

### 2.4 K2 deficiency stimulates apoptosis of anchorage‐dependent cells

One of the major differences between anchorage‐dependent and ‐independent cells is their expression of the Kindlin family members. In contrast to K3, which is exclusively expressed in anchorage‐independent cells, K2 is expressed primarily in anchorage‐dependent cells such as fibroblasts, endothelial cells and muscle cells. Thus, we sought to characterize the function of K2 in anchorage‐dependent cells.

**FIGURE 5:**
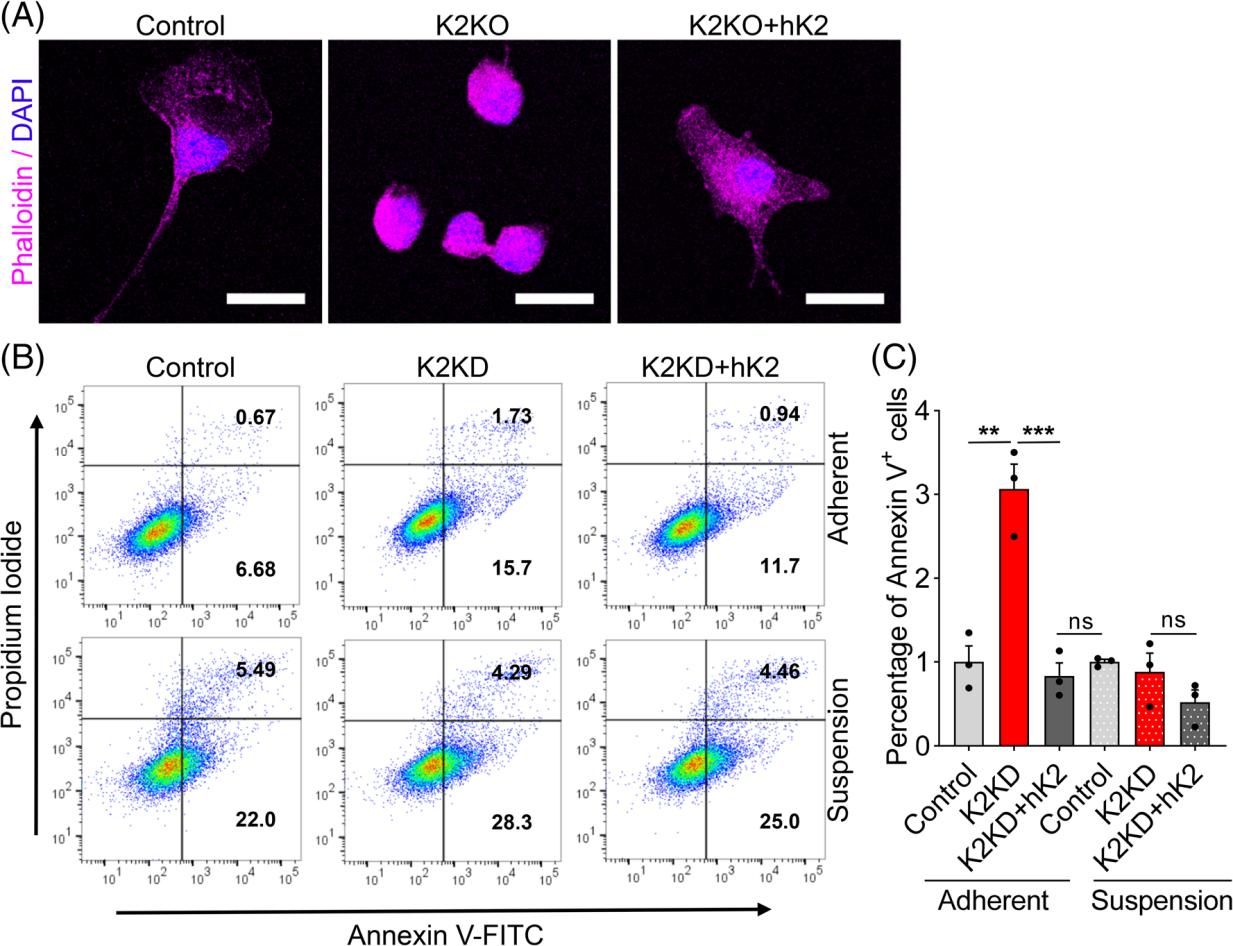
K2 knockout leads to apoptosis of anchorage‐dependent cells. (A) Representative confocal images of control, K2KO (K2 knockout by CRISPR‐Cas9), and K2KO + hK2 (K2 knockout with re‐expression of human K2) mouse embryonic fibroblasts (MEFs) stained with Phalloidin. Note that the loss of K2 expression was accompanied by morphological changes (stretched to round) which were reversed by re‐expression of human K2. Scale bars are 20 μm. (B) Flow cytometry analysis of Annexin V expression by control, K2KD (K2 knockdown by siRNA) and K2KD + hK2 (K2 knockdown with re‐expression of human K2) MEFs in adherent or suspension cultures. Numbers represent the percentages of cells in the corresponding quadrants. (C) Quantification of Annexin V^+^ apoptotic cells in B. *N* = 4. All values are mean ± SEM. Statistical significance was determined by one‐way ANOVA.

K2 knockout (K2KO) was achieved by CRISPR‐Cas9 gene editing in mouse embryonic fibroblasts (MEFs). The absence of Kindlin2 protein was confirmed by Western blot analysis (Figure S1A,B). The deletion of K2 in MEFs completely terminates cell adhesion which is rescued by the re‐expression of human K2 (hK2) in MEFs (K2KO + hK2) (Figure 5A). We knocked down K2 expression in MEFs by siRNA interference to create the K2 knockdown (K2KD) MEFs with a reduced ~75% Kindlin2 protein level (Figure S1C,D). The defective K2 expression in K2KD MEFs resulted in cell apoptosis as shown by increased Annexin V expression in K2KD MEFs, which was reversed by re‐expression of hK2 in MEFs (K2KD + hK2) (Figure 5B,C). These effects were only observed in adherent cells suggesting that promotion of apoptosis by K2 deficiency is anchorage‐dependent.

### 2.5 K2 deficiency inhibits proliferation of anchorage‐dependent cells in a K2‐PXN interaction‐dependent manner

**FIGURE 6:**
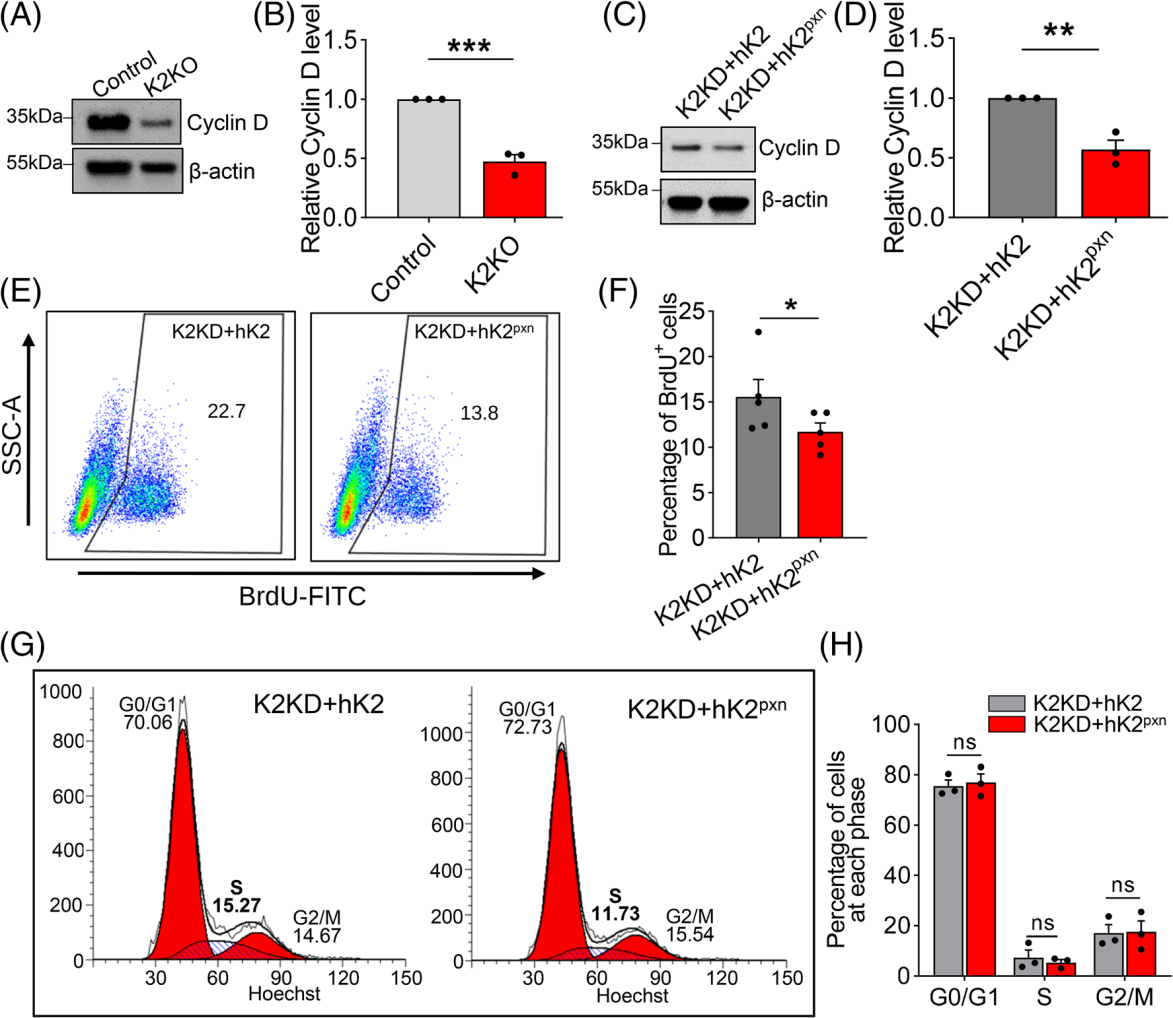
Inhibition of cell proliferation by K2 knockout is dependent upon K2‐PXN interaction. (A) Western blot analyses of Cyclin D protein expression in control and K2KO MEFs. (B) Bar graph showing a significant decrease in Cyclin D protein expression in K2KO MEFs compared with controls. *N* = 3. (C) Western blot analysis of Cyclin D protein expression in K2KD + hK2 and K2KD + hK2^pxn^ MEFs. K2KD + hK2^pxn^ represents K2KD MEFs with re‐expression of human K2 carrying mutations at the PXN‐binding site. (d) Quantification of western blots in C showing decreased Cyclin D protein expression in K2KD + hK2^pxn^ MEFs. *N* = 3. (E) Flow cytometry analysis of BrdU incorporation by K2KD + hK2 and K2KD + hK2^pxn^ MEFs. Numbers indicate percentages of cells in each quadrant. (F) Quantification of flow cytometry analysis in E showing significantly fewer BrdU^+^ cells in the K2KD + hK2^pxn^ group. *N* = 3. (G) Flow cytometry cell cycle analysis of K2KD + hK2 and K2KD + hK2^pxn^ MEFs. Numbers represent the percentages of cells in each phase. (H) Quantification of cell cycle analysis in G. *N* = 3. All values are mean ± SEM. Statistical significance was determined by Student's *t*‐test (B, D and F) or two‐way ANOVA (H).

Both K3 and K2 are required for cell adhesion but their deficiencies affect apoptosis in opposite ways. K2 deficiency suppresses MEF proliferation in contrast to the promotion of proliferation by K3 deficiency, as Cyclin D was downregulated in K2KO MEFs compared with controls (Figure 6A,B). This effect relies upon the interaction between K2 and PXN as K2KD + hK2^pxn^ (K2KD MEFs with re‐expression of hK2 carrying mutations at the PXN‐binding site) MEFs expressed lower Cyclin D compared with K2KD + hK2 (Figure 6C,D). Flow cytometry analysis of BrdU incorporation demonstrated that K2KD + hK2^pxn^ MEFs had fewer proliferating cells compared with K2KD + hK2 (Figure 6E,F), which supports our conclusion that the inhibitory effect on proliferation of anchorage‐dependent cells by K2 deficiency depends upon the K2‐PXN interaction. Our flow cytometry cell cycle analyses did not reveal any significant difference (Figure 6G,H), probably because the residual normal K2 in the K2KD cells counterbalanced the defective hK2^pxn^.

## 3 DISCUSSION

Here we identify the molecular interaction between integrin binding partners with opposing roles in anchorage‐dependent and ‐independent cells, which underlies paradoxical functions previously assigned to integrin adaptors. Two orthologs of the Kindlin family, K2 and K3, are characteristic for adhesomes of adherent and non‐adherent cells, respectively, and both are equally required for cell adhesion. Unexpectedly, they exhibit opposing effects on cell apoptosis and proliferation. K2 promotes survival and proliferation of anchorage‐dependent cells, while K3 suppresses survival and proliferation of anchorage‐independent cells. Similarly, the effect on proliferation is dependent upon the Kindlin‐PXN interaction, regardless of Kindlin type or anchorage dependence.

Integrin‐mediated survival and dependence on the ECM are known as common mechanism that operates in all cells to prevent their misplacement and abnormal growth in the wrong location.^1^, ^41^ This mechanism, however, does not apply to circulating haematopoietic cells thriving in anchorage‐independent conditions despite sharing the very same components of adhesomes with professional adherent cells.^1^, ^41^ We show that while the presence and engagement of K2 in anchorage‐dependent cells enables their survival, K3 in haematopoietic cells has a completely opposite role. First, deletion of K3 promotes anchorage‐independent cell survival and proliferation. Second, disruption of K3 binding to its partner cytoskeletal ‘clutch’ PXN, is sufficient to promote anchorage‐independent growth. On the contrary, K2 linkage to PXN is required for adherent cell survival and proliferation. These conclusions are supported by the detailed structural analysis of the K2‐PXN interaction using point mutations disrupting their binding site.^42^ K2‐deficient fibroblasts featured inhibited proliferation and abnormally upregulated apoptosis in our study, which is a reminder of the defective integrin‐deficient embryoid body exhibiting smaller size, lower proliferation rate and irregular shape compared with controls.^43^ Similar to our observations, K2 deficiency has been reported to induce apoptosis and restrain proliferation of other anchorage‐dependent cells, including chondrocytes,^44^ smooth muscle cells,^45^ mesenchymal stromal cells^46^ and so on. The behaviour of the K2‐PXN mutant, at least in part, resembled both K2 and PXN knockouts, characterized by impaired cell adhesion, spreading and migration.^47^, ^48^, ^49^ Collectively, K2 and its binding to PXN serve as a ‘keeper’ of cell adhesiveness, and associated cell survival for anchorage‐dependent cells.

Surprisingly, in contrast to K2KO cells, K3 knockout cells behaved in a completely opposite way to integrin knockout cells.^43^ Cellular characteristics of K3 deficiency resembled those of transformed cells. K3‐deficient human lymphoma cells grew faster and survived better in our transplantation experiment. K3‐deficient macrophages exhibited increased proliferation and survival against apoptosis. In contrast to adherent cells, the proliferation of haematopoietic cells was not dictated by adhesion, since increased proliferation was observed in non‐adherent K3‐deficient cells as well as in K3^pxn^ mutants where adhesion was not preserved. Another distinction between K2 and K3 is their activation state required for function. Because adhesion to ECM is required for the proper growth of anchorage‐dependent cells, K2 is constitutively activating integrins in anchorage‐dependent cells. However, haematopoietic cells thrive without attachment to the ECM which means that integrins are deactivated most of the time unless they are immobilized in blood clots or on the surface of inflamed endothelium^5^, ^6^, ^7^ when integrins are activated by K3. Thus, it appears that the connections between K3 and its binding partners restrict cell survival and proliferation in non‐adherent cells, which is on the opposite side of K2.

K3 deficiency in humans could cause severe bleeding, immune incompetence and osteoporosis, attributable to integrin activation defects on haematopoietic cells including platelets and leukocytes.^36^ Our study demonstrates that K3 deficiency promotes growth and reduces apoptosis of human tumour cells and that K3 deficiency protects against anoikis of anchorage‐independent cells which favours tumour metastasis.^2^ These results echo a recent study identifying K3 as a novel tumour suppressor.^16^ K3 is significantly downregulated in several tumours by gene hypermethylation and deletions, and tumour cells dramatically increase their metastasis after K3 knockdown.^16^ Exiting the original growing milieu and implanting it in other organs is an important property of haematopoietic tumours (e.g., lymphoma). An anchorage dependence switch could happen in this process, that is, from circulating in the blood in an anchorage‐independent state to in situ growing in organs (i.e., spleen) in an anchorage‐dependent state, whether this process is accompanied by a switch in Kindlin expression pattern is still unknown.

In conclusion, we show that K3 deficiency promotes human lymphoma growth. While K2 and its binding to PXN protect anchorage‐dependent cells from the loss of adhesion and subsequent anoikis, the very same interaction in K3 expressing anchorage‐independent cells serves as a suppressor of protumorigenic processes by restricting their proliferation, survival and cell adhesion.

## 4 METHODS AND MATERIALS

### 4.1 Cells

Raw 264.7 macrophages (ATCC TIB‐71) and MEF cells (ATCC SCRC‐1008) were purchased from ATCC. Cells were cultured in DMEM medium (Gibco) supplemented with 10% fetal bovine serum (FBS; Gibco) and 50 μg/ml penicillin/ streptomycin (Gibco). K3KO, K3KO + hK3 and K3KO + hK3^pxn^ raw 264.7 macrophages have been described elsewhere.^39^ K3KO, K3^hypo^ and K3^int^ microglia were isolated from corresponding mice. Primary microglia were isolated from the brains of postnatal day 1 mice as described previously.^50^ Briefly, the cerebral hemispheres were harvested and disassociated in cold PBS, and the brain suspension was filtered through a 70‐μm sieve and centrifuged to pellet the cells. The cell pellet was then resuspended in DMEM/F12 (with 20% FBS, 100 U/ml penicillin and streptomycin, 0.25 μg/ml amphotericin B and supplemented with non‐essential amino acids) and plated onto plastic dishes. After 2–4 weeks, the floating microglia cells were harvested from the conditional media by centrifugation at 700 g for 10 min. K2KO, K2KO + hK2, K2KD and K2KD + hK2 MEFs were described previously.^39^, ^42^ Human peripheral blood lymphocytes expressing normal K3 and deficient K3 were described previously.^36^ Human peripheral blood lymphocytes were immortalized by transfection with EBV. These cells were cultured in RPMI 1640 medium supplemented with 15% FBS (Gibco) and 50 μg/ml penicillin/streptomycin (Gibco). Experiments involving human cells were approved by the Institutional Review Board at the Cleveland Clinic and conducted in accordance with the ethical standards of the Institutional Review Board at the Cleveland Clinic and the Helsinki Declaration of 1975, which was revised in 2000. Informed consent was obtained from all individuals. More details about the patients' info were published previously.

### 4.2 Immunofluorescent staining

Cells were seeded on fibronectin pre‐coated coverslips for 1 h, then washed with cold PBS, fixed with 4% paraformaldehyde for 15 min, permeabilized with 0.1%Triton X‐100 for 5 min, blocked in blocking solution (3% bovine serum albumin and 10% goat serum in PBS) at room temperature for 1 h, and incubated with primary antibodies at 4°C in blocking solution overnight. On the second day, cells were washed with PBS and then incubated with secondary antibodies at room temperature for 1 h. Finally, cells were mounted onto slides with mounting medium (Vector Laboratory H‐2000‐10). Confocal images were obtained with a Leica confocal microscope and analysed with the FIJI Image J software.

The primary antibodies used were: Phalloidin (Thermofisher Scientific A‐21245 and A12381), Ki67 (Millipore AB9260), cleaved Caspase 3 (Cell Signalling Technology 9661S), Cyclin D1 (92G2 (Cell Signalling Technology 2978) and wheat germ agglutinin (WGA, Thermofisher Scientific W32466).

### 4.3 Animals

The K3^hypo^ and K3^int^ mice were reported previously.^39^ To create the myeloid‐specific inducible K3 knockout (K3KO) mouse line, K3^f/f^ mice (exon 2 of the K3 gene is flanked by 2 loxP sites) were crossed with CX3CR1‐Cre (tamoxifen‐inducible, the Jackson Laboratory) mice. Nude mice (007850) and SCID mice (001303) were commercially available from the Jackson Laboratory. Animal experimental procedures were performed in accordance with National Institutes of Health (NIH) guidelines on animal care and all protocols were approved by the Institutional Animal Care and Use Committee of the Cleveland Clinic.

### 4.4 Flow cytometry, cell cycle analysis and apoptosis analysis

Flow cytometry buffer (PBS without Ca^2+^ or Mg^2+^, 1 mM EDTA, 25 mM HEPES pH 7.0 and 1% heat‐inactivated FBS) was used to wash and resuspend cells. BrdU incorporation assay and apoptosis analysis were conducted using FITC BrdU Flow Kit (BD Biosciences 559619) and FITC Annexin V Apoptosis Detection Kit (BD Biosciences 556547) following the manufacturer's protocol. For cell cycle analysis, cells were cultured for 12 h and then incubated with 20 μg/ml Hoechst 33342 for 1.5 h. After incubation, cells were washed and sent for analysis by a BD flow cytometer. The flow data were analysed by the Flow Jo software.

### 4.5 Western blot analysis

Cells were lysed with 1% SDS lysis buffer and resolved on 10% precast protein gel (Bio‐Rad Laboratories 4561036). Proteins were then transferred to PVDF membranes (Millipore). Membranes were blocked with 5% nonfat dry milk in TTBS (0.2 M Tris pH 7.4, 1.5 M NaCl, 0.1% thimerosal and 0.5% Tween 20) and incubated with primary antibodies overnight at 4°C followed by HRP‐conjugated secondary antibodies. Protein expression was detected by ECL Detection Reagent (Thermofisher Scientific 32106). Images were captured by ChemiDoc (Bio‐Rad Laboratories) or on film.

The following antibodies were used for western blotting: GAPDH (Cell Signaling Technology D16H11), cleaved PARP (Cell Signaling Technology 9544T), K3 (Abcam ab68040), K2 (Millipore MAB2617), HA‐Tag (Cell Signaling Technology 3724S), rabbit IgG (Cell Signaling Technology 7074S) and mouse IgG (Cell Signaling Technology 7076S).

### 4.6 Human lymphoma model in nude mice

For subcutaneous injections, mice were anaesthetised with ketamine (40 mg/kg)/xylazine (7.5 mg/kg). Approximately 6 million cells per injection were inoculated subcutaneously into the hind flanks. Following inoculation, tumour length and width were measured daily when they reached a visible size. The subcutaneous tumours and organs were harvested after euthanasia. For intraperitoneal injections, we used the same number of cells. The experimental animals were monitored daily over 12 weeks for signs of tumour development (e.g., abdominal distension and/or lethargy) or changes in their overall health status (e.g., rough coat, severe weight loss, or difficulties in eating and drinking or with ambulation). Organs were collected after euthanasia. Cells in the abdominal flush were fixed, stained for EBV antigen or human IgG and sent for flow cytometry analysis.

### 4.7 Statistics

All data are expressed as mean ± SEM. Student's *t*‐tests were performed to compare two groups. For multiple groups, ANOVA followed by appropriate post hoc analysis was used to determine statistical differences. A *p*‐value <0.05 was considered significant. *p <0.05, ** *p* <0.01 and *** *p* <0.001.

## AUTHOR CONTRIBUTIONS

Irina Zhevlakova performed the experiments, interpreted the data and wrote the manuscript. Luyang Xiong wrote the manuscript. Tejasvi Dudiki and Alieta Ciocea performed the experiments and interpreted the data. Huan Liu performed the experiments. Eugene Podrez conceived the project and design the experiments. Tatiana V. Byzova conceived the project, design the experiments, interpreted the data, wrote the manuscript and secured the funding.

## CONFLICT OF INTEREST

The authors declare no potential conflict of interest.

## Supporting information


**Figure S1** Generation of K2‐deficient cells. (A) Schematic of the generation of K2KO MEFs and re‐expression of hK2. K2KO MEFs were generated by CRISPR‐Cas9 and then re‐expressed vector (Vec) (Pcmv‐HA‐vector) or human K2 (Pcmv‐HA‐hK2) or human K2 with mutants in Paxillin‐binding site (hK2^pxn^) (Pcmv‐HA‐hK2‐G42K/L46E), respectively by lipofectamine 3000 transfection. (B) Western blot analysis of mouse K2 (mK2) and human K2 (hK2) protein expression in K2KO and K2KO + hK2 MEFs. (C) Schematic of the generation of K2KD MEFs and re‐expression of hK2. K2KD MEFs were generated by siRNA interference and then re‐expressed vector (Vec) (Pcmv‐HA‐vector) or hK2 (Pcmv‐HA‐hK2) or hK2^pxn^ (Pcmv‐HA‐hK2‐G42K/L46E), respectively. (D) Western blot analysis of mK2 and hK2 protein expression in K2KD and K2KD + hK2 MEFs.

## Data Availability

No codes or sequencing datasets were generated in this study. All materials and protocols will be available upon request.
