# Supplementary material for: Opposite roles of Kindlin orthologs in cell survival and proliferation
Source: Cell Prolif. 2022 Jul 20;55(9):e13280. doi: 10.1111/cpr.13280 (PMC9436913; doi:10.1111/cpr.13280)
Supplement: Supplementary file 1 — Figure S1 Generation of K2‐deficient cells. (A) Schematic of the generation of K2KO MEFs and re‐expression of hK2. K2KO MEFs were generated by CRISPR‐Cas9 and then re‐expressed vector (Vec) (Pcmv‐HA‐vector) or human K2 (Pcmv‐HA‐hK2) or human K2 with mutants in Paxillin‐binding site (hK2pxn) (Pcmv‐HA‐hK2‐G42K/L46E), respectively by lipofectamine 3000 transfection. (B) Western blot analysis of mouse K2 (mK2) and human K2 (hK2) protein expression in K2KO and K2KO + hK2 MEFs. (C) Schematic of the generation of K2KD MEFs and re‐expression of hK2. K2KD MEFs were generated by siRNA interference and then re‐expressed vector (Vec) (Pcmv‐HA‐vector) or hK2 (Pcmv‐HA‐hK2) or hK2pxn (Pcmv‐HA‐hK2‐G42K/L46E), respectively. (D) Western blot analysis of mK2 and hK2 protein expression in K2KD and K2KD + hK2 MEFs. [file CPR-55-e13280-s001.docx]

Supplementary figure 1

CRISPR Cas9 K2 knockout

a

**K2**


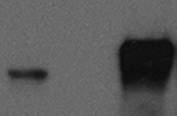

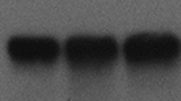


35kd

40kd

100kd

70kd

GAPDH

HA-hK2

mK2

Control+Vec

K2KO+Vec

K2KO+hK2

siRNA K2 Knockdown

b

c

100kd

35kd

40kd

70kd

GAPDH

HA-hK2

mK2


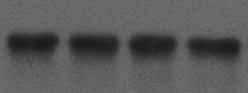

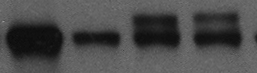


NC+Vec

K2KD+Vec

K2KD+hK2

K2KD+hK2^pxn^


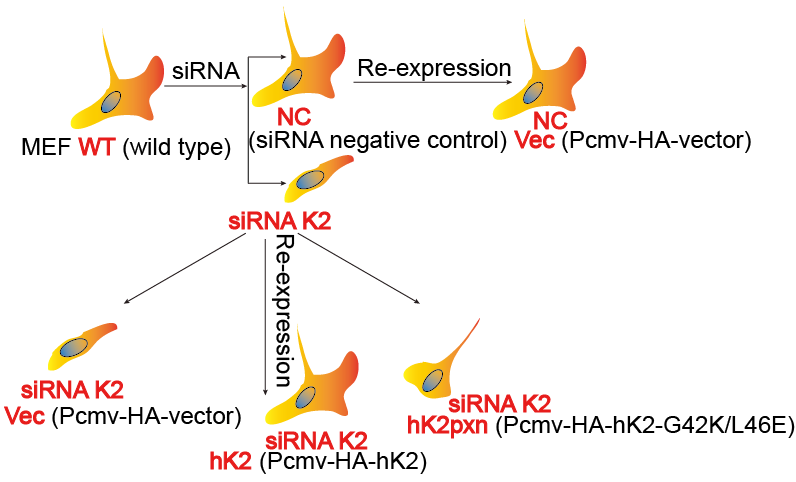


**hK2^pxn^**


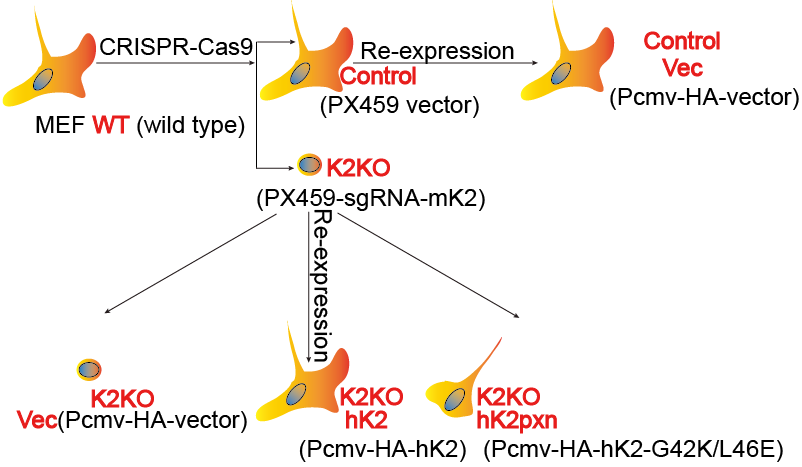


**hK2^pxn^**

d
